# Developing an Accurate and Fast Non-Destructive Single Leaf Area Model for Loquat (*Eriobotrya japonica* Lindl) Cultivars

**DOI:** 10.3390/plants8070230

**Published:** 2019-07-17

**Authors:** Maurizio Teobaldelli, Youssef Rouphael, Giancarlo Fascella, Valerio Cristofori, Carlos Mario Rivera, Boris Basile

**Affiliations:** 1 Department of Agricultural Sciences, University of Naples Federico II, 80055 Naples, Italy; 2 CREA Research Centre for Plant Protection and Certification, 90011 Bagheria (Palermo), Italy; 3 Department of Agriculture and Forest Sciences, Tuscia University, 01100 Viterbo, Italy

**Keywords:** Indirect measurement, plant phenotyping, leaf shape, model calibration, bootstrap, validation

## Abstract

In this research, seven different models to predict leaf area (LA) of loquat (*Eriobotrya japonica* Lindl) were tested and evaluated. This species was chosen due to the relevant importance of its fruit as an appreciated early summer product and of its leaves and flower as a source of additional income within the nutraceutical and functional food markets. The analysis (calibration and validation) was made using a large dataset (2190) of leaf width (W), leaf length (L), and single LA collected in ten common loquat cultivars. During the analysis, the results obtained using one- and two-regressor models were also evaluated to assess the need for fast measurements against different levels of accuracy achieved during the final estimate. The analysis permitted to finally select two different models: 1) a model based on a single measurement and quadratic relationship between the single LA and W (*R^2^* = 0.894; root mean squared error [RMSE] = 12.98) and another model 2) based, instead, on two measurements (L and W), and on the linear relationship between single LA and the product of L × W (*R^2^* = 0.980; RMSE = 5.61). Both models were finally validated with an independent dataset (cultivar ‘Tanaka’) confirming the quality of fitting and accuracy already observed during the calibration phase. The analysis permitted to select two different models to be used according to the aims and accuracy required by the analysis. One, based on a single-regressor quadratic model and W (rather than L) as a proxy variable, is capable of obtaining a good quality of fitting of the single LA of loquat cultivars (*R^2^* = 0.894; RMSE = 12.98), whereas, the other, a linear two-regressor (i.e., W and L) model, permitted to achieve the highest prediction (*R^2^* = 0.980; RMSE = 5.61) of the observed variable, but double the time required for leaf measurement.

## 1. Introduction

Leaves are one of the fundamental physiological hubs of a plant in which processes, such as photosynthesis and transpiration, take place. Together with other plant apparatus (e.g., xylem strands and phloem elements), leaves contribute to the absorption of nutrients and water from the soil through the roots and allow the translocation of photosynthetic products from the source to the sink organs. Moreover, within a life cycle of a plant, leaves pass from a juvenile heterotrophic state to a mature autotrophic condition [1] determining source-to-sink transitions [2].

Previous research carried out in tomatoes showed that a higher leaf area (LA) after fruits set can increase fruit size at harvest [3]. This latter aspect is quite important considering that for some species (e.g., *Prunus* sp.) large fruit size, pulp firmness, and sweetness are considered important fruit quality traits [4]. For instance, as reported by Kappel et al. [4], fruit size at harvest of *Prunus* sp. is highly dependent on the relationships between yield efficiency, leaf area (LA), and crop load. Moreover, higher rates of fruit drop might arise after reduction in LA (e.g., due for instance to herbivores, defoliation, and leaf shading) which causes a decrease of available resources needed for fruit development [5].

For this reason, the analysis of LA in fruit trees is considered of utmost importance and models are needed to estimate this parameter rapidly and accurately. On the other hand, considering that the analysis of plant parameters during the entire cycle of growth might be tedious, it is important to clearly define the objective of the research [6] and the methodologies to be used. For instance, LA can be measured directly (1) by destructive harvest of leaves and the subsequent measurement of their area in the laboratory obtained generally using a planimeter, digital LA meter, or image processing [7], or (2) by non-destructive measurements directly on the plant, using a portable digital LA meter, or (3) indirectly taking other proxy measurements (e.g., leaf length and width) and allometric approaches. Direct methods are generally simple and accurate although the measurements involved can be laborious and time-consuming making it difficult to get a representative spatial sample [8] and making large-scale implementation only marginally feasible [9]. In addition, the great spatial and temporal variability of canopies structure makes the direct measurement not really suitable for long-term monitoring of LA development. Moreover, direct measurement of LA, carried out through the excision of leaves might damage the canopy—especially if frequent measurements of LA are needed during the growing season; therefore, affecting by non-negligible uncertainty, other measurements that may need to be carried out on the same plant [10,11]. Indirect methods, in which LA is inferred from observations of other proxy variables directly on the plant, such as the leaf length (L) and leaf width (W) [12], are generally faster, amenable to automation, and thereby, allow a larger spatial sample to be obtained [8].

So far, several models and regression equations have been proposed, either to be applied at the leaf [11,13] or shoot level [14,15,16,17,18,19,20,21,22,23]. Until now, models and regression equations for fruit trees cover several species, like for instance, apricot [24], avocado [25], banana [26], blackberry [25], cacao [27], chestnut [28], citrus [29], grapevine [14,15,20,25,30,31,32,33], guava [34], hazelnut [18], kiwifruit [25,35], lotus plum [25], mango [36], medlar [37], passion fruit [38], peach [39], persian walnut [40], persimmon [25,41], pistachio [42], rabbiteye blueberry [43], red currant [25], small fruits [44], red raspberry [25], sour orange [45], strawberry [46,47], pecan [48], sweet cherry [49,50], and white mulberry [51]. In this context, it seems interesting to also analyse the loquat (*Eriobotrya japonica* Lindl), a fruit tree species belonging to the *Rosaceae* family.

Loquat is a subtropical evergreen perennial fruit shrub or small tree, preferably cultivated in light, well-drained, deep (>1.5 m), moist, alluvial soils, with mild climate (on average air temperature >15 °C and less than 25 °C after fruit set), with few wind (fruit can be damaged), no risk of frost (−12 °C is fatal for the plants, but preferably higher than −5 °C to avoid damage to the fruits), and with rainfalls (650–1000 mm annually) well-distributed throughout the year. Currently, China is the main producer, (1,000,000 tons produced annually on 170,000 ha) followed by Spain (40,000 tons annually), Pakistan (30,000 tons annually), and Turkey (10,000–20,000 tons) [52]. In Italy, with a production of about 6000 tons, it is listed among the minor fruit trees covering a total area of 450 hectares [53], generally represented by family orchards and isolated trees. As the current management are considered sometimes too rudimentary, new data and information on this species can help to cultivate it in a more rational manner, especially considering that loquat might represent, with its anti-inflammatory and diuretic properties, promising cultivation of the future. Loquat fruits have a juicy, nutritious, and palatable pulp and can be used to make jam, wine (together with seeds that are rich in starch), fruit juice, syrup, and candied fruit [54]. They are generally much appreciated by the consumers especially because fruits are available in the market out of season, in early summer [54]. Moreover, loquat plants, with their heavy fragrance, are also nectariferous, with a great honey potential [54]. Moreover, thanks to the presence of volatile oils (e.g., farnoquiol) and vitamin B17, loquat leaves have a notable curative effect against coughs and asthma and lung cancer [54,55]. Finally, a recent study [56] carried out on loquat cultivar (cv.) Golden Nugget reports the presence of a high concentration of phenolic bioactive products in loquat leaves and flower, especially during the green stage, (i.e., chlorogenic acid and quercetin derivatives) with antioxidant capacity and inhibitory activity against enzymes relevant for hyperglycemia such as α-amylase and α-glucosidase. Therefore, the two products, together with fruits, may constitute a source of additional income within the nutraceutical and functional food markets [56].

Therefore, considering the importance of this species, this paper aims: (1) to test and compare rapid (i.e., based on quick measurements such as L and W), but still accurate, generalised allometric models for loquat species that can be used for different cultivars, and (2) to validate, using an independent data set and rigorous statistical analysis, the best models.

## 2. Results and Discussion

### 2.1. Leaf Data Analysis of the Tested Cultivars

In this study, seven generalised allometric models for retrieving, rapidly and accurately, single LA of loquat cultivars were tested, compared, and validated using an independent data set. The importance of this analysis is related to the unquestioned importance represented by leaf architecture within the broad fields of botany, plant physiology, and fruit crops. The photosynthetic rate per unit area is strongly influenced by leaf morphology (size, shape, symmetry, venation, organisation, and petiole characteristics) and leaf cell anatomy (cell types and their size, shape, density, and the size and distribution of intercellular air spaces) [57]. However, although leaf thickness and the internal architecture is fundamental for maximising light collection and carbon dioxide uptake, the ability to intercept light is dependent only on its two-dimensional structure, i.e., L and W [58].

For this reason, many studies still highlight the importance of studying LA of several fruit crops, so that it would be possible to characterise leaf functions and structures, based only on those two important proxy variables [13,24,40,44,59,60,61,62].

In our study, the analysis of the training set (constituted by pooled leaf area values measured from nine different cultivars of *Eriobotrya japonica* in 2015) permitted to evaluate how different morphometric characteristics, such as L and W, were related to the single leaf area. Specifically, L ranged between 10.0 and 33.3 cm, whereas W ranged between 2.5 and 12.1 cm (Table 1).

At the same time, single LA values ranged between 25.35 and 274.00 cm^2^. All those values should be intended as the range of utilisation of the proposed seven models. Another parameter considered in this study was the aspect ratio of the leaf (i.e., length to width ratio or L:W) [63]. In Angiosperms, the leaf shape is an important characteristic regulated by several genetic factors and mutations which testify how leaves cope with their environment [58] and whose diversity can be, often, discussed in functional and evolutionary terms of natural selection [64]. Indeed, although a larger total area is obviously more advantageous in terms of photosynthetic productivity, there are cases (e.g., flooding areas) in which the presence of wider leaves might also constitute a disadvantage [65]. Besides these aspects, the collected leaf aspect ratio data can be useful to carry out leaf phenotyping of loquat cultivars and as part of a training database, to train machine learning algorithms to classify cultivars using shape features, as has already happened, for instance, with leaves of several species [66] and cultivar identification of fruits [67]. In loquat, the mean leaf shape ratio of the training set was equal to 3.25 (SE = ±0.54) with pooled data ranging from a minimum of 1.91 and a maximum of 6.12 (Table 2), meaning that on average, the polar diameter exceeded the equatorial diameter more than once (i.e., an elliptical leaf shape). Overall, the maximum mean leaf shape ratio corresponded to the cv. ‘Precoce di Palermo’ (mean = 3.804 ± 0.52) whereas the minimum leaf shape ratio was related to the cv. ‘Nespolone di Trabia’ (mean = 2.941 ± 0.28) (Table 2). The data of the training and validation sets collected during the 2015 and 2016 growing seasons, respectively were quite similar and, of particular importance, the data (cv. ‘Tanaka’) utilised to validate the models were always comprised within the range of utilisation of the calibrated models (Table 1). The mean leaf shape ratio value of loquat estimated in this study was similar to that published for the Chinese litchi and higher than that reported for other common fruit crops (Table 3 [68]).

### 2.2. Model Calibration and Validation

In the current study, linear and exponential (Table 4) models, based on one regressor (L or W; models 1 to 6), and used to estimate the desired variable (LA) showed a good fitting (*R^2^* ranged between 0.836 and 0.880) but compared to the other models utilised in this research, a moderate accuracy root mean squared error [RMSE] ranged from 13.82 to 16.14 cm^2^). Instead, a better accuracy (*R^2^* was ranging between 0.853 and 0.894; RMSE ranged from 12.98 to 15.27 cm^2^) was obtained by using a quadratic relationship between L or W and the dependent variable (LA) (Table 4).

**Figure 1:**
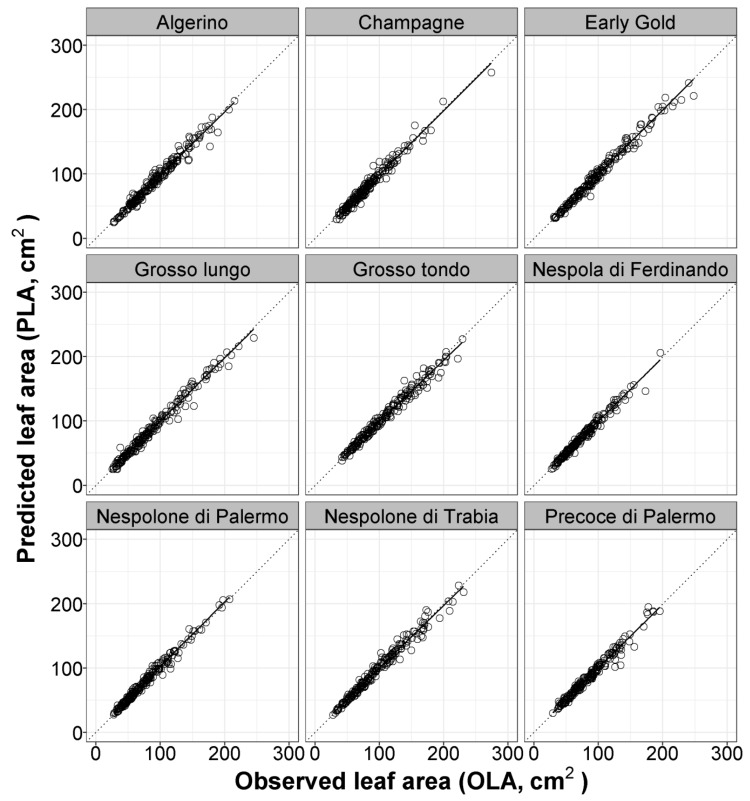
Plots of predicted leaf area (PLA) using model 7 [LA = −0.516 + 0.667 × (L × W)], obtained with pooled data of nine different loquat cultivars, versus observed values of single leaf areas (OLA) of each cultivar used in the calibration experiment (data collected during 2015). Dotted lines represent the 1:1 relationship between the predicted and observed values. The solid line represents the linear regression line of each model.

In general, to estimate a single LA of loquat cultivars the use of W should be preferred as a predictor in linear, exponential, and quadratic modelling. In fact, as showed by the analysis, the best ranking (bayesian information criterion [BIC] = 14,854 ÷ 15,089; predicted residual error sum of squares [PRESS] = 314,867 ÷ 357,115; sum of squared errors [SSE] = 313,948 ÷ 356,091) and better fitting and accuracy (*R^2^* = 0.880 ÷ 0.894; RMSE = 12.98 ÷ 13.83) (Figure 1) was achieved by using as regressor W, rather than L (*R^2^* = 0.836 ÷ 0.853; RMSE = 15.27 ÷ 16.14; BIC = 15,458 ÷ 15,664; PRESS = 435,447 ÷ 486,356; SSE = 434,241 ÷ 484,990). In particular, by comparing the six different single-regressor allometric equations, model 6 was the best ranking and; therefore, to estimate the single LA of loquat cultivars using only one measurement, W and bootstrapped coefficients of model 6 (Table 5) should be preferred.

Compared to the single regressor models, a better predictive capability to estimate the single LA of loquat cultivars was reached by using the product (L × W) of the two proxy variables (i.e., L and W). In fact, as showed by the ranking (BIC = 11,732; PRESS = 58,879; SSE = 58,694) and accuracy (*R^2^* = 0.980; RMSE = 5.614) values, model 7 ranked first between all the models tested in this study (Table 4).

**Figure 2:**
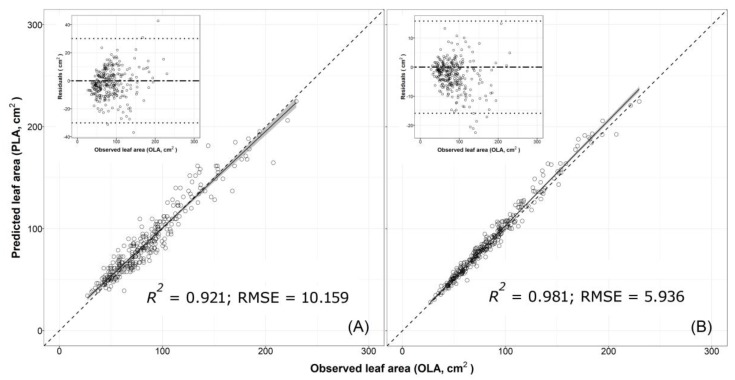
Plot of predicted leaf area (PLA) estimated using (**A**) one-regressor bootstrapped model 6 [LA = 14.003 + 1.742 × W2] and (**B**) two-regressors bootstrapped model 7 [LA = −0.526 + 0.667 × (L × W)] versus observed values of single leaf areas (OLA) of cv. ‘Tanaka’ collected during 2016 (validation experiment). The solid line and the grey area represent, respectively, linear regression lines of the bootstrapped models 6 and 7 and generalised linear smoothing. *R^2^* and root mean squared error (RMSE) are also reported. Dotted lines represent the 1:1 relationship between the predicted and observed values. The analysis of the dispersion pattern of residuals for models 6 and 7 are shown in the insets. Residuals = the difference between predicted leaf areas (PLA) estimated by model 6 or 7 (with coefficients obtained from pooled data from nine loquat cultivars, see Table 4 for more details) versus the observed leaf area of ‘Tanaka’ cultivar sampled in 2016 (validation experiment). The solid line is the mean of the differences. The broken lines are the limits of agreement, calculated as d ± 3 SD (standard deviation); where d is the mean of the differences, and SD is the standard deviation of the differences. If the differences are normally distributed, 97% of the differences in a population will lie between the limits of agreement.

Finally, considering that the shape of a loquat leaf is essentially similar to an ellipse (whose area is generally calculated as the product of Pi (π) times the semi-major axis of length L times the semi-minor axis of length W), and as expected, the product of L × W gave the best fitting of the single LA. Therefore, following the above considerations, to accurately estimate single LA of loquat cultivars both L and W measurements are necessary (Figure 2) and the bootstrapped coefficients of model 7 can be used (Table 5).

Regarding the selection of the best model to be utilised to estimate loquat single LA, a balance should be searched between the performances of the model itself and the number of variables (the economy) needed to make the prediction [20,72]. Generally, simple and convenient equations that only involve one variable have to be preferred [72]. On the other hand, it should also be noted that although the time required for the leaf measurement is doubled, models that require two measurements per leaf, generally estimate single LA accurately [24,25,73]. Therefore, the decision about what kind of model should be used (one or two regressors) depends mainly on the aim of the study and the desired accuracy of the estimates.

## 3. Materials and Methods

### 3.1. Experimental Site and Plant Material

The experiment was carried out in 2015 and 2016 at the experimental station of the Council for Agricultural Research and Economics (CREA) located in Caserta, southern Italy on mature loquat trees. All trees were grafted in the year 1990 onto seedling rootstocks and trained to an open-vase shape and with a tree spacing of 5 m × 5 m (400 plants ha^−1^).

The trial included a total of nine cultivars used for model calibration (‘Algerino’, ‘Champagne’, ‘Early Gold’, ‘Grosso Lungo’, ‘Grosso Tondo’, ‘Nespola di Ferdinando’, ‘Nespolone di Palermo’, ‘Nespolone di Trabia’, ‘Precoce di Palermo’) measured in 2015 and the cv. ‘Tanaka’ used for model validation (data collected during 2016).

### 3.2. Data Collection

A total of 210 healthy leaves were collected for each of the nine cultivars used for model calibration (the calibration dataset; therefore, consisted of 1890 leaves), whereas 300 leaves were collected for the cultivar ‘Tanaka’. The leaves were rapidly transported to the lab, where the following parameters were individually measured on each sample leaf: L, W, and LA of the leaf blade. LA was measured with an LA meter (LI-3100; LICOR, Lincoln, NE, USA) calibrated to 0.01 cm^2^.

### 3.3. Statistical Analysis

#### 3.3.1. Model Calibration

The choice of the allometric models to be used was based on the specific morphometric characteristics and leaf shape traits (in this case length: width ratio or L:W; [63]) of the leaves of *Eriobotrya japonica*. In the genus *Eriobotrya*, leaves are alternate, simple, coriaceous, coarsely dentate, and with a short petiole. Usually, loquat’s leaves have a shape similar to an ellipse (L: 12–30 cm; W: 3–9 cm) with a shining upper lamina and, often, a lower pubescent surface [74].

The estimation of the LA of nine loquat cultivars was; therefore, carried out on pooled data (training set; *n* = 1890; Table 1) using seven different regression models (Table 4), of which three were linear, two quadratic, and two exponential. The allometric models were essentially based on one fast measurement of a proxy variable (i.e., the L or W of the leaves) or the product of the previous two variables (L × W).

Fitting of the linear and quadratic models was made in the statistical software R-STAT [75] using the lm function available in the CRAN (comprehensive R archive network) Stats package in, whereas the exponential models were fitted in R-STAT using the nonlinear least-squares minimisation (nlsLM) function available in the CRAN package minpack.lm [76] (Table 4).

The performance of the seven different allometric models were based on the following criteria: highest R-squared (*R²*), lowest RMSE, lowest BIC [77], lowest PRESS criterion [78,79], lowest SSE, and the lowest bias (i.e., the evidence of the internal validity of the fitted model; [80] between the PRESS and SSE values. The BIC criteria and PRESS were both computed in R-STAT using the functions available, respectively, in the CRAN packages Stats [75] and in the modelling and analysis of real-time PCR data (qpcR) [81]. Finally, once the best ranking model was chosen, the coefficients of the selected models were tuned using the non-parametric bootstrapping function available in the CRAN package Boot [82]. The bootstrapped model coefficients together with the associated, standard error, median, and per cent confidence intervals were obtained iteratively in 1000 bootstrapped samples selected with the replacement of observations from the original data set (*n* = 1890).

#### 3.3.2. Model Validation

Validation of the selected bootstrapped models was made by comparing the predicted values of a single LA estimated using proxy values (L and W) available in the validation set (*n* = 300) of cv. ‘Tanaka’ measured during a field experiment carried out in 2016 and the observed single LA values. In this case, the goodness of fit of the selected models was based on the R-squared (*R²*) and the RMSE of the observed LA (OLA) versus predicted LA (PLA) of ‘Tanaka’ loquat cultivar.

## 4. Conclusions

Based on the previous results, if the objective of the analysis is to carry out numerous, continuous (such us during the entire cycle of vegetative growth), and fast estimates of the single LA of loquat cultivars, measurements should be preferably based on a single predictor, and in particular on W rather than on L. Therefore, once measurements of the W parameter are obtained from a consistent sample of plants, a quadratic model (model 6, Table 4) should be chosen and used to estimate the value of interest (i.e., single LA) with a good quality of fitting (*R^2^* = 0.894) and moderate accuracy (RMSE = 12.98).

On the other hand, if the analysis requires better accuracy, a two-regressor model should be preferred and, accordingly, measurements of both L and W carried out. So doing, it will be possible to achieve the highest prediction (*R^2^* = 0.980; RMSE = 5.614) of the single LA of loquat cultivars.

In conclusion, considering the importance of loquat species, with leaves and fruit constituting a likely source of additional income within the nutraceutical and functional food markets [56], this study and the proposed allometric models can represent an important resource to better evaluate growth stages, productivity, treatment to pest, and pathogen effectiveness or to indirectly estimate other important indicators such as leaf area index (LAI), a value generally used in combination with gas exchange and chlorophyll fluorescence measurements.

## Figures and Tables

**Table 1:** Characteristics of predictors (L, W) and dependent variable (LA) used in this study.

Group	No. of Cultivars	No. of Leaves Sampled	L (cm)			W (cm)			L × W (cm²)			L:W Mean (± SE)	LA (cm²)		
			Min	Max	Mean	Min	Max	Mean	Min	Max	Mean		Min	Max	Mean
Training set	9	1890	10	33.3	20.17	2.5	12.1	6.341	38	387.2	133.28	3.25 (0.47)	25.35	274	88.4
Validation set	1	300	12.6	31.8	20.27	3.4	11	6.268	42.84	337.7	131.66	3.29 (0.54)	27.3	229.7	84.61

L: leaf length; W: leaf width; LA: leaf area; L:W: length to width ratio (leaf shape ratio); SE: standard error.

**Table 2:** Characteristics of the leaf shape ratio (L:W) from different cultivars used in this study.

Cultivar	Leaf Shape Ratio (L:W)			
	Max	Mean	Standard Deviation	Min
Algerino	4.349	2.947	0.357	1.910
Champagne	4.677	3.276	0.411	2.067
Early Gold	4.943	3.092	0.451	2.250
Grosso lungo	6.120	3.726	0.643	2.540
Grosso tondo	5.167	3.472	0.435	2.291
Nespola di Ferdinando	4.400	2.981	0.361	1.984
Nespolone di Palermo	4.016	3.014	0.309	2.250
Nespolone di Trabia	3.864	2.941	0.281	2.238
Precoce di Palermo	5.270	3.804	0.524	2.339
Tanaka	5.231	3.293	0.467	2.176
Total	6.120	3.256	0.528	1.910

**Table 3:** Comparison of mean values of leaf shape ratio of Loquat with other values reported for other fruit crops in the literature.

Species	L:W	References
Apple	1.76	[69]
Apricot	1.14	[24]
Chinese litchi	3.19	[68]
Citrus	1.85	[29]
Durian	2.42	[70]
Hazelnut	1.23	[18]
Loquat	3.25	This study
Medlar	2.38	[37]
Mulberry	2.71	[71]
Persimmon	1.45	[41]

**Table 4:** Fitted constant (*a*) and coefficient (*b*) of the models used to estimate the loquat leaf area (LA in cm^2^) of single leaves from leaf length (L) and leaf width (W) measurements. The standard errors and *p*-value in parenthesis; L and W were in cm. All data were derived from the calibration Experiment 2015 (*n* = 1890 leaves).

Model No.	Form of the Model Tested	Fitted Coefficient and Constant		*R^2^*	RMSE	BIC	PRESS	SSE	Bias (PRESS-SSE)
		*a* (SE/*p*-Value)	*b* (SE/*p*-Value)						
1	LA = *a* + *b* × L	−83.292 (1.801/***)	8.510 (0.087/***)	0.836	16.14	15,664	486,356	484,990	1366
2	LA = *a* × e*^b^*^×L^	14.610 (0.291/***)	0.086 (0.001/***)	0.851	15.74	15,571	462,944	461,346	1598
3	LA = *a* + *b* × L^2^	2.227 (0.901/*)	0.203 (0.002/***)	0.853	15.27	15,458	435,447	434,241	1206
4	LA = *a* + *b* × W	−61.081 (1.321/***)	23.575 (0.202/***)	0.880	13.83	15,089	357,115	356,091	1024
5	LA = *a* × e*^b^*^×W^	19.217 (0.284/***)	0.230 (0.002/***)	0.878	13.82	15,087	357,303	355,669	1634
6	LA = *a* + *b* × W^2^	14.025 (0.666/***)	1.741 (0.014/***)	0.894	12.98	14,854	314,867	313,948	919
7	LA = *a* + *b* × (L × W)	−0.516 (0.321/ns)	0.667 (0.002/***)	0.980	5.614	11,732	58,879	58,694	185

Note: *** *p* < 0.001; * *p* < 0.05; ns = not significant; *R^2^* = coefficient of determination; RMSE (cm^2^) = root mean squared error; BIC = Bayesian information criterion, PRESS = predicted residual error sum of squares; SSE = sum of squared error; Bias = differences between the PRESS and SSE values.

**Table 5:** Main outputs for non-parametric bootstrap analysis (replications: 1000) of models 6 and 7 fitted with data from the loquat single leaf area (LA in cm^2^) from leaf length (L) and width (W) measurements. Standard errors and *p*-value in parenthesis; L and W were in cm.

Model N.	Dependent Variable	Number of Predictor Variables	Parameter	Original		Boot						Percent Confidence Interval	
				Value	(*p*-Value)	Value	Bias	SE	Med	Skew	Kurtosis	2.5%	97.5%
6	LA	1	*R^2^*	0.894	-	0.894	0.000	0.005	-	-	-	-	-
			RMSE	12.980	-	12.992	−0.012	2.704	-	-	-	-	-
			(intercept)	14.025	(***)	14.003	−0.022	0.706	14.021	−0.013	−0.335	12.656	15.395
			W^2^	1.741	(***)	1.742	0.001	0.018	1.742	−0.038	−0.405	1.707	1.778
7	LA	2	*R^2^*	0.980	-	0.980	3.24 × 10^−5^	0.001	-	-	-	-	-
			RMSE	5.614	-	5.6022	−0.012	1.334	-	-	-	-	-
			(intercept)	−0.516	(ns)	−0.526	−0.011	0.350	−0.525	0.013	0.301	−1.211	0.176
			L × W	0.667	(***)	0.667	0.000	0.003	0.667	0.048	0.202	0.661	0.673

Note: *** *p* < 0.001; ns = not significant; *R^2^* = coefficient of determination; RMSE (cm^2^) = root mean squared error.
